# Practice effects in medical school entrance testing with the undergraduate medicine and health sciences admission test (UMAT)

**DOI:** 10.1186/1472-6920-14-48

**Published:** 2014-03-12

**Authors:** Ian B Puddey, Annette Mercer, David Andrich, Irene Styles

**Affiliations:** 1 Faculty of Medicine, Dentistry and Health Sciences, University of Western Australia, 35 Stirling Hwy, Crawley, WA 6009, Australia; 2 Graduate School of Education, University of Western Australia, 35 Stirling Hwy, Crawley, WA 6009, Australia

## Abstract

**Background:**

The UMAT is widely used for selection into undergraduate medical and dental courses in Australia and New Zealand (NZ). It tests aptitudes thought to be especially relevant to medical studies and consists of 3 sections – logical reasoning and problem solving (UMAT-1), understanding people (UMAT-2) and non-verbal reasoning (UMAT-3). A substantial proportion of all candidates re-sit the UMAT. Re-sitting raises the issue as to what might be the precise magnitude and determinants of any practice effects on the UMAT and their implications for equity in subsequent selection processes.

**Methods:**

Between 2000 and 2012, 158,909 UMAT assessments were completed. From these, 135,833 cases were identified where a candidate had sat once or more during that period with 117,505 cases (86.5%) having sat once, 14,739 having sat twice (10.9%), 2,752 thrice (2%) and 837, 4 or more times (0.6%). Subsequent analyses determined predictors of multiple re-sits as well as the magnitude and socio-demographic determinants of any practice effects.

**Results:**

Increased likelihood of re-sitting the UMAT twice or more was predicted by being male, of younger age, being from a non-English language speaking background and being from NZ and for Australian candidates, being urban rather than rurally based. For those who sat at least twice, the total UMAT score between a first and second attempt improved by 10.7 ± 0.2 percentiles, UMAT-1 by 8.3 ± 0.2 percentiles, UMAT-2 by 8.3 ± 0.2 percentiles and UMAT-3 by 7.7 ± 0.2 percentiles. An increase in total UMAT percentile score on re-testing was predicted by a lower initial score and being a candidate from NZ rather than from Australia while a decrease was related to increased length of time since initially sitting the test, older age and non-English language background.

**Conclusions:**

Re-sitting the UMAT augments performance in each of its components together with the total UMAT percentile score. Whether this increase represents just an improvement in performance or an improvement in understanding of the variables and therefore competence needs to be further defined. If only the former, then practice effects may be introducing inequity in student selection for medical or dental schools in Australia or NZ.

## Background

Practice effects to improve performance in tests of cognitive ability are well established [1] but the magnitude and determinants of such effects may well vary according to the test. The Undergraduate Medicine and Health Sciences Admission Test (UMAT) is a cognitive ability test designed and delivered by the Australian Council for Educational Research (ACER) on behalf of the UMAT Consortium of Australian and New Zealand universities [2]. It has been devised to determine aptitude for study in medicine, dentistry and other courses for health professionals. The rapid expansion and increasing popularity of commercially available preparation courses for the UMAT [3] has resulted in a reciprocal increase in interest in the potential magnitude and determinants of any practice effects that may be improving test performance.

The UMAT is not a test of personality, values or attitudes, nor is it a measure of previous learning in academic domains. It comprises three sections: UMAT-1 - Logical Reasoning and Problem Solving, UMAT-2 - Understanding People and UMAT-3 - Non-verbal Reasoning and is a 3-hour paper-and-pencil test. Each section is assessed using multiple choice questions with 4 or 5 optional responses, of which only one is correct. The three sections in 2012 comprised 48 items over 70 minutes in UMAT-1, 44 items over 55 minutes in UMAT-2 and 44 items over 55 minutes in UMAT-3. In Section 1 (UMAT-1) candidates are required to exercise logical reasoning and problem solving skills using both inductive and deductive reasoning, with an emphasis on logical argument in working to a solution. In Section 2 (UMAT-2) the emphasis is on assessing empathy and emotional intelligence with candidates required to show an understanding of the thoughts, feelings, behaviour and intentions portrayed within each question. Section 3 (UMAT-3) evaluates a candidate’s non-verbal reasoning skills and aims to measure cognitive ability independent of language ability and specific cultural knowledge. ACER provides example questions in an information booklet and informs potential candidates that some practice in answering questions of a similar type, and under similar test conditions to those in the actual test, may be a useful preparation. They also provide two practice tests online for candidates who are registered to sit the UMAT. Whether preparation or actual coaching for the test makes a difference to test scores, however, remains controversial [3,4].

Practice effects for the UMAT may be of substantial magnitude as recently reported in a relatively small study from New Zealand [4]. In that study results from 263 students who sat the UMAT both in 2010 and 2011 were analysed. Approximately 85% of the cohort improved their overall score, 11% decreased and 4% obtained exactly the same score. The mean increase averaged approximately 9.3% for the total score, 9.4% for UMAT-1, 7% for UMAT-2 and 10.6% for UMAT-3. When considered in terms of relative percentile score the study cohort improved from a median total UMAT score percentile of 52% on the first attempt to 73% on the second. ACER in their annual reports have also consistently reported an increase in standardised scores in each section of the UMAT in those who sit in consecutive years [2]. In 2012 they reported that the majority of students who were re-sitting the test improved their scores but with estimated mean increases that were lower than those seen in the NZ study (UMAT-1, 6.4%, UMAT-2, 5.5% and UMAT-3, 3.1%).

As a cognitive ability test, the construct validity and predictive validity of the UMAT have been subjected to recurrent careful analysis [5,6]. If practice effects are indeed substantive they could potentially influence either the construct validity or predictive validity of the test or both. We have therefore ascertained all cases between 2000 and 2012 where candidates re-sat the UMAT and now report the magnitude of the practice effects observed on re-testing and discuss some of their possible determinants.

## Methods

Between 2000 and 2012, 158,909 UMAT assessments were completed. After generating a unique ID for each candidate based on name, date of birth and gender 135,833 cases were identified where a candidate had sat once or more during that period with 117,505 cases (86.5%) having sat once, 14,739 having sat twice (10.9%), 2,752 thrice (2%) and 837, 4 or more times (0.6%). Subsequent analyses determined predictors of multiple re-sits as well as the magnitude and socio-demographic determinants of any practice effects.

Practice effects were examined in relation to total UMAT percentile scores and the percentile scores in each of its 3 subsections - UMAT-1 - Logical Reasoning and Problem Solving, UMAT-2 - Understanding People and UMAT-3 - Non-verbal Reasoning. Each year ACER reports candidate results as scores on a separate scale for each of UMAT-1, UMAT-2 and UMAT-3 with an overall UMAT score calculated as the average of these three scores. Over the years 2000 to 2012, the scale has changed several times as the test has developed. As a result scores are not necessarily comparable between years. However, percentile ranks enable a measure of the relative standing of a candidate within each cohort. Given the large numbers in each cohort it is likely that there is reasonable comparability in the competence of candidates at specified percentile ranks across cohorts. Hence the use of percentile ranks as the measure in this study.

The practice effects were further evaluated by dividing the cohort into quartiles of initial test performance and analysing the upper and lower quartiles in relation to number of times the UMAT was sat. Predictors of the magnitude of any practice effects were evaluated from a number of socio-demographic indices collected on enrolment for the UMAT, including age, gender, postal address, language spoken at home, type of secondary school, country of origin and self-identification as being of Aboriginal or Torres Strait Islander origin (ATSI). Language spoken at home was classified according to the Australian Standard Classification of Languages (ASCL), 2011 [7]. For multivariate analysis this was collapsed into 4 groups – English, European languages, Asian languages and all other languages. Type of secondary school was divided into one of 5 groups – government (publicly funded), independent (fee paying), Catholic, Technical and Further Education institutions (TAFE – public provider of predominantly vocational tertiary education courses) and Other. For those with an Australian address, an index of rurality was generated by linking each candidate’s postcode to the Accessibility/Remoteness Index of Australia (ARIA) [8]. ARIA values are grouped into one of five categories: Highly Accessible (ARIA score 0–1.84), Accessible (ARIA score >1.84 - 3.51), Moderately Accessible (ARIA score >3.51 -5.80), Remote (ARIA score >5.80 - 9.08) and Very Remote (ARIA score >9.08 – 12).

The project has been approved by the Human Research Ethics Committee at the University of Western Australia (file reference RA/4/1/2178).

### Statistics

The magnitude of change between the first test and first re-sit were compared by paired T-tests. Comparisons of scores between those who sat once versus those who re-sat the test were made by unpaired T-tests. Univariate comparisons of performance during multiple re-sits of the UMAT were made by repeated measures analysis of variance (with post-hoc comparisons by Bonferroni correction). Multivariate analyses utilised linear regression to assess the independent relationships of change in total UMAT, UMAT-1, UMAT-2 and UMAT-3 score between the first and second tests with age, gender, type of secondary school, language spoken at home, country of origin, ARIA score and self-identification as ATSI as independent predictors. Logistic regression analysis was utilised to ascertain potential independent predictors of the likelihood of sitting the UMAT more than once. All analyses were carried out utilising IBM SPSS Statistics Version 20.0.

## Results

### Socio-demographic data

Of the 135,833 cases where a candidate had sat once or more during the period 2000 to 2012, 1.1% were aged less than or equal to 16 yr, 35.6% were 17 yr, 43.3% were 18 yr, 8.4% were 19 yr, 9.9% were aged 20 to 30 yr and 1.7% were greater than 30 yr. Females comprised 57.8% of all cases and males 42.2%. English was spoken at home by 69.2%, Asian languages by 27.1%, European languages by 2% and other languages by 1.7%. School of origin was a government school for 47%, independent school for 32.8%, Catholic school for 17.6%, TAFE college for 0.4% and Other school for 2.2%. The majority of the population were Australian (86.9%) with 12.1% from New Zealand and 0.9% from other countries. Only 0.4% self-declared as ATSI in origin. For Australian residents (N = 118,086), 93.4% were living in Highly Accessible areas, 4.9% in Accessible areas, 1.2% in Moderately Accessible areas, 0.4% in Remote areas and 0.1% in Very Remote areas. The socio-demographic profile by year in which the UMAT was sat is outlined in Additional file 1. It demonstrates an increasing number of 18 and 19 yo sitting the test over time with a decreasing number of those either 17 yo or less or those greater than 20 yo sitting the test. The proportion of females has decreased slightly while that of males has increased. The proportion of those from an Asian language background has almost doubled from 18% in 2001 to 32% in 2012. The proportion from a government school background has slightly increased while those from an independent or Catholic school background has commensurately decreased. The numbers of New Zealand cases increased substantially from 2005 onwards with introduction of the UMAT as a selection tool for the 2 NZ medical schools, but have remained relatively steady since at approximately 13-15% of the cohort.

### Magnitude of practice effects

**Figure 1:**
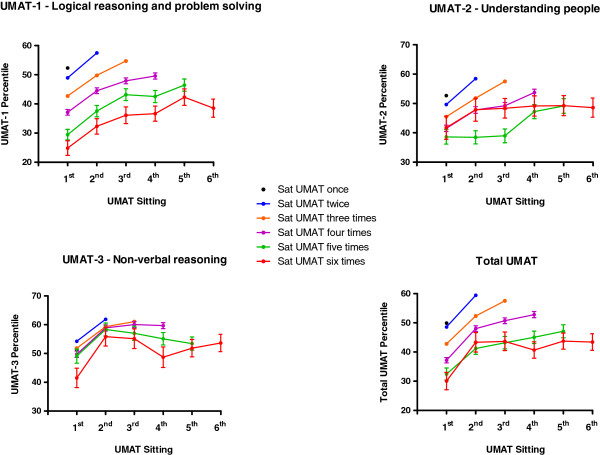
**Total UMAT percentile score and percentile score in each of its 3 subsections (± SEM) by number of times candidates re-sat the test between 2000 and 2012.** Error bars are absent where they are too small relative to the mean.

The changes in total UMAT percentile score and each of its subsections are depicted in Figure 1. The predominant increment for total UMAT occurred between the first attempt and the first re-sit. RANOVA indicated a further significant increase in percentile score at the second re-sit (P < 0.001, Bonferroni correction) but no further increment with subsequent re-sits. For UMAT-1 further increases in relative performance extended out to the 4^th^ re-sit (P = 0. 024 compared to the 4^th^ re-sit, Bonferroni correction). For UMAT-2 increases in relative percentile extended to only the 3^rd^ re-sit (P = 0. 001 compared to the 2^nd^ re-sit, Bonferroni correction) while for UMAT-3 there was only an increase in relative percentile between the first attempt and the first re-sit with no further increment at any subsequent re-sit.

For those who sat at least twice (N = 18,328), the total UMAT percentile score between a first and second attempt improved in 71.1%, decreased in 28.7% and was identical in 0.2% of candidates. Total UMAT score improved by a mean of 10.7 ± 0.2 percentiles (Paired T-test P < 0.001). The UMAT-1 percentile score improved in 66.6%, decreased in 33.2% and was identical in 0.2% of candidates with a mean increase of 8.3 ± 0.2 percentiles (Paired T-test P < 0.001). The UMAT-2 percentile score improved in 62.6%, decreased in 37.3% and was identical in 0.1% of candidates and increased overall by 8.3 ± 0.2 percentiles (Paired T-test P < 0.001). The UMAT-3 percentile score improved in 61.1%, decreased in 38.7% and was identical in 0.2% of candidates with a mean overall increase of 7.7 ± 0.2 percentiles (Paired T-test P < 0.001).

**Figure 2:**
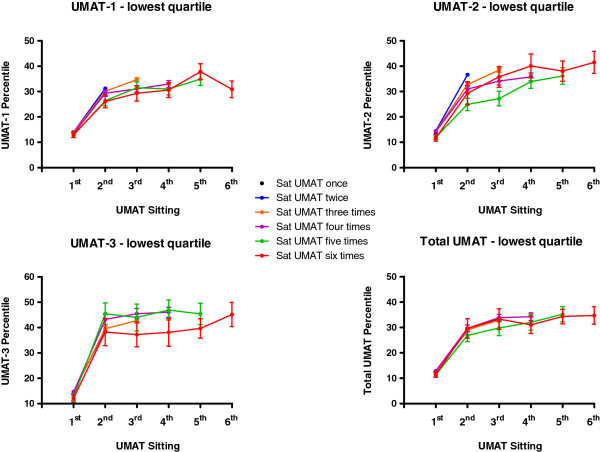
**Total UMAT percentile score and percentile score in each of its 3 subsections (± SEM) by number of times candidates re-sat the test between 2000 and 2012 for those in the lowest performance quartile in their first test.** Error bars are absent where they are too small relative to the mean.

**Figure 3:**
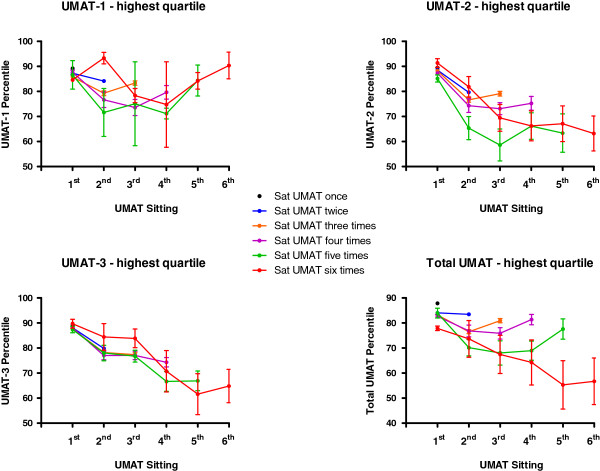
**Total UMAT percentile score and percentile score in each of its 3 subsections (± SEM) by number of times candidates re-sat the test between 2000 and 2012 for those in the highest performance quartile in their first test.** Error bars are absent where they are too small relative to the mean.

Total UMAT percentile score and percentile score in each of its 3 subsections by number of times candidates re-sat the test between 2000 and 2012 are depicted for those in the lowest performance quartile in their first test in Figure 2 and for those in the highest performance quartile in Figure 3. In those in the lowest quartile of initial performance the increases were by 16.2 ± 0.3 percentiles for total UMAT score (Paired T-test P < 0.001), 16.8 ± 0.3 percentiles for UMAT-1 (Paired T-test P < 0.001), 21.1 ± 0.3 percentiles for UMAT-2 (Paired T-test P < 0.001) and 24.8 ± 0.4 percentiles for UMAT-3 (Paired T-test P < 0.001). For those in the highest quartile of initial performance there were decreases by 1.4 ± 0.3 percentiles for total UMAT score (Paired T-test P < 0.001), 3.6 ± 0.3 percentiles for UMAT-1 (Paired T-test P < 0.001), 9.3 ± 0.3 percentiles for UMAT-2 (Paired T-test P < 0.001) and 8.5 ± 0.3 percentiles for UMAT-3 (Paired T-test P < 0.001).

### Predictors of magnitude of practice effects

Linear regression analyses of potential predictors of the change in overall performance in total UMAT percentile score and each of its sub-sections on re-testing are outlined in Tables 1, 2, 3 and 4. Performance in UMAT-1, UMAT-2 and UMAT-3 were first forced into the model to correct for regression to the mean. Lower percentile scores in UMAT-1, UMAT-2 or UMAT-3 predicted higher increments in each of these scores on re-testing as anticipated. Separate models (data not shown) indicated that for those in the lowest quartile of total UMAT percentile score at initial testing, the predicted increment on re-testing was 18.8 percentiles (CI 18.0, 19.7). For those in the lowest quartile of UMAT-1 it was 22.5 percentiles (CI 21.6, 23.4), for those in the lowest quartile of UMAT-2 it was 33.5 percentiles (CI 32.5, 34.6) and for those in the lowest quartile of UMAT-3 it was 35.7 percentiles (CI 34.7, 36.7). The number of years between the first test and the first re-sit was then forced into the model, adjusting for a predicted negative association between time interval and any measured practice effects. The predicted negative association was highly significant for UMAT-2 and UMAT-3 but was not seen for UMAT-1 performance. Together these predictors accounted for 9.7% of the variance in performance between the first and second tests in the total UMAT, 12.7% in UMAT-1, 18.5% in UMAT-2 and 24.5% in UMAT-3. Finally, the socio-demographic predictors were entered into the model. The significant predictors of an increase in overall performance were being male or being a candidate from New Zealand rather than from Australia, while performance on re-sit was lower for older candidates and for those from Asian language, European language or Other language backgrounds compared to an English language background. Together these predictors only increased the variance for the full model for total UMAT to 12.2%, UMAT-1 to 15.3%, UMAT-2 to 22.7% and UMAT-3 to 27% (Tables 1, 2, 3 and 4). The increase in performance for those from NZ was primarily driven by an increase in the UMAT-3 percentile score while the decrease in percentile scores in those with a non-English language background was not evident for UMAT-3 in those from Asian languages background (Table 4).

**Table 1:** **Multivariate linear regression for change in total UMAT score from first sit to second sit (N = 17,089, r**^**2**^ **= 0.122)**

***Predictor variable (reference group in brackets)***	**N**	**B (95% CI)**	**Beta**	**P-value**
***UMAT Score Percentile Test 1***	17089	-0.26 (-0.27, -0.25)	-0.343	**<0.001**
***Number of years between tests***	17089	-0.88 (-1.14, -0.62)	-0.048	**<0.001**
***Age*** *(≤ 16 yr of age)*	222			
*17 yr*	5958	-0.81 (-3.27, 1.66)	-0.020	0.522
*18 yr*	7209	-2.20 (-4.66, 0.26)	-0.055	0.080
*19 yr*	1682	-6.02 (-8.62, -3.41)	-0.091	**<0.001**
*20 - 30 yr*	1749	-8.91 (-11.49, -6.33)	-0.138	**<0.001**
*> 30 yr*	269	-10.18 (-13.47, -6.89)	-0.065	**<0.001**
***Gender*** *(Females)*	9463			
*Males*	7626	0.62 (0.06, 1.17)	.016	**0.030**
***Language spoken at home*** *(English)*	10726			
*Asian Languages*	5611	-4.32 (-4.94, -3.70)	-0.103	**<0.001**
*European Languages*	349	-2.48 (-4.45, -0.51)	-0.018	**0.014**
*Other Languages*	403	-6.91 (-8.76, -5.07)	-0.053	**<0.001**
***School type*** *(Government)*	8911			
*Catholic*	2565	-0.63 (-1.46, 0.20)	-0.011	0.137
*Independent*	5102	0.92 (0.26, 1.57)	0.021	**0.007**
*Other*	84	-3.03 (-4.82, -1.23)	-0.024	**0.001**
*TAFE*	427	-2.04 (-6.05, 1.96)	-0.007	0.317
***Country*** *(Australia)*	12522			
New Zealand	4436	1.22 (0.53, 1.91)	0.027	**0.001**
Other Country	131	2.32 (-0.86, 5.50)	0.010	0.153
***Aboriginal & Torres Strait Islander*** *(ATSI)*	17044			
*Non-ATSI*	45	-4.99 (-10.38, 0.39)	-0.013	0.069

(Significant P-values are boldfaced).

**Table 2:** **Multivariate linear regression for change in UMAT-1 score from first sit to second sit (N = 17,089, r**^**2**^ **= 0.153)**

***Predictor variable *** **( *reference group in brackets *)**	**N**	**B (95% CI)**	**Beta**	**P-value**
***UMAT-1 Score Percentile Test 1***	17089	-0.31 (-0.32, -0.30)	-0.399	**<0.001**
***Number of years between tests***	17089	-0.06 (-0.33, 0.22)	-0.003	0.686
***Age*** (≤ *16 yr of age*)	222			
*17 yr*	5958	-1.82 (-4.38, 0.75)	-0.042	0.165
*18 yr*	7209	-3.01 (-5.57, -0.45)	-0.072	**0.021**
*19 yr*	1682	-6.14 (-8.85, -3.43)	-0.088	**<0.001**
*20* - *30 yr*	1749	-9.91 (-12.60, -7.22)	-0.144	**<0.001**
>*30 yr*	269	-12.25 (-15.68, -8.83)	-0.073	**<0.001**
***Gender*** (*Females*)	9463			
*Males*	7626	2.38 (1.79, 2.96)	0.057	**<0.001**
***Language spoken at home*** (*English*)	10726			
*Asian Languages*	5611	-3.48 (-4.13, -2.83)	-0.079	**<0.001**
*European Languages*	349	-3.17 (-5.22, -1.13)	-0.022	**0.002**
*Other Languages*	403	-5.33 (-7.26, -3.41)	-0.039	**<0.001**
***School type*** (*Government*)	8911			
*Catholic*	2565	-1.51 (-2.37, -0.65)	-0.026	**0.001**
*Independent*	5102	0.45 (-0.24, 1.13)	0.010	0.203
*Other*	84	-2.54 (-4.41, -0.67)	-0.019	**0.008**
*TAFE*	427	-2.32 (-6.49, 1.85)	-0.008	0.275
***Country*** (*Australia*)	12522			
New Zealand	4436	0.74 (0.02, 1.45)	0.015	**0.045**
Other Country	131	1.41 (-1.91, 4.72)	0.006	0.405
***Aboriginal & Torres Strait Islander*** (*ATSI*)	17044			
*Non*-*ATSI*	45	-2.80 (-8.40, 2.81)	-0.007	0.328

(Significant P-values are boldfaced).

**Table 3:** **Multivariate linear regression for change in UMAT-2 score from first sit to second sit (N = 17,089, r**^**2**^ **= 0.227)**

***Predictor variable *** **( *reference group in brackets *)**	**N**	**B (95% CI)**	**Beta**	**P-value**
***UMAT-2 Score Percentile Test 1***	17089	-0.31 (-0.32, -0.30)	-0.494	**<0.001**
***Number of years between tests***	17089	-0.06 (-0.33, 0.22)	0.033	**<0.001**
***Age*** (≤ *16 yr of age*)	222			
*17 yr*	5958	-1.82 (-4.38, 0.75)	-0.004	0.884
*18 yr*	7209	-3.01 (-5.57, -0.45)	-0.028	0.352
*19 yr*	1682	-6.14 (-8.85, -3.43)	-0.041	**0.031**
*20* - *30 yr*	1749	-9.91 (-12.60, -7.22)	-0.068	**<0.001**
>*30 yr*	269	-12.25 (-15.68, -8.83)	-0.014	0.161
***Gender*** (*Females*)	9463			
*Males*	7626	2.38 (1.79, 2.96)	-0.082	**<0.001**
***Language spoken at home*** (*English*)	10726			
*Asian Languages*	5611	-3.48 (-4.13, -2.83)	-0.170	**<0.001**
*European Languages*	349	-3.17 (-5.22, -1.13)	-0.022	**0.001**
*Other Languages*	403	-5.33 (-7.26, -3.41)	-0.066	**<0.001**
***School type*** (*Government*)	8911			
*Catholic*	2565	-1.51 (-2.37, -0.65)	-0.008	0.262
*Independent*	5102	0.45 (-0.24, 1.13)	0.025	**0.001**
*Other*	84	-2.54 (-4.41, -0.67)	-0.024	**0.001**
*TAFE*	427	-2.32 (-6.49, 1.85)	-0.006	0.361
***Country*** (*Australia*)	12522			
New Zealand	4436	0.74 (0.02, 1.45)	-0.010	0.181
Other Country	131	1.41 (-1.91, 4.72)	0.003	0.612
***Aboriginal & Torres Strait Islander*** (*ATSI*)	17044			
*Non*-*ATSI*	45	-2.80 (-8.40, 2.81)	-0.008	0.227

(Significant P-values are boldfaced).

**Table 4:** **Multivariate linear regression for change in UMAT-3 score from first sit to second sit (N = 17,089, r**^**2**^ **= 0.270)**

***Predictor variable *** **( *reference group in brackets *)**	**N**	**B (95% CI)**	**Beta**	**P-value**
***UMAT-3 Score Percentile Test 1***	17089	-0.49 (-0.50, -0.48)	-0.525	**<0.001**
***Number of years between tests***	17089	-3.13 (-3.44, -2.82)	-0.130	**<0.001**
***Age*** (≤ *16 yr of age*)	222			
*17 yr*	5958	0.11 (-2.83, 3.06)	0.002	0.939
*18 yr*	7209	-1.09 (-4.03, 1.85)	-0.021	0.467
*19 yr*	1682	-6.33 (-9.44, -3.22)	-0.073	**<0.001**
*20* - *30 yr*	1749	-9.72 (-12.80, -6.63)	-0.115	**<0.001**
>*30 yr*	269	-11.99 (-15.93, -8.06)	-0.058	**<0.001**
***Gender*** (*Females*)	9463			
*Males*	7626	3.65 (2.98, 4.33)	0.071	**<0.001**
***Language spoken at home*** (*English*)	10726			
*Asian Languages*	5611	-0.54 (-1.27, 0.19)	-0.010	0.145
*European Languages*	349	-3.28 (-5.63, -0.93)	-0.018	**0.006**
*Other Languages*	403	-4.40 (-6.59, -2.20)	-0.026	**<0.001**
***School type*** (*Government*)	8911			
*Catholic*	2565	-1.61 (-2.60, -0.62)	-0.022	**0.001**
*Independent*	5102	1.01 (0.22, 1.80)	0.018	**0.012**
*Other*	84	-3.28 (-5.43, -1.14)	-0.020	**0.003**
*TAFE*	427	-3.94 (-8.72, 0.84)	-0.011	0.106
***Country*** (*Australia*)	12522			
New Zealand	4436	1.89 (1.07, 2.72)	0.032	**<0.001**
Other Country	131	3.06 (-0.74, 6.86)	0.010	0.114
***Aboriginal & Torres Strait Islander*** (*ATSI*)	17044			
*Non*-*ATSI*	45	-5.25 (-11.68, 1.19)	-0.010	0.110

(Significant P-values are boldfaced).

### Predictors of re-sitting the UMAT

When comparing those who sat once versus those who re-sat, the percentile scores in UMAT-1 (52.3 ± 0.09 vs 47.4 ± 0.20, Unpaired T-test, P < 0.001) and UMAT-2 (52.7 ± 0.09 vs 48.6 ± 0.21, Unpaired T-test, P < 0.001) were initially lower in those who re-sat while those in UMAT-3 were initially higher (51.2 ± 0.8 vs 53.7 ± 0.20, Unpaired T-test, P < 0.001). The results from binomial logistic regression of predictors of the likelihood of sitting the UMAT twice or more are outlined in Table 5. For those in the lowest quartile for UMAT-1 or UMAT-2 percentile score at the first test, the likelihood of re-sitting was increased (OR 2.09, 95%CI 1.97, 2.23, P < 0.001, and OR 1.24, 95% CI 1.17, 1.31, P < 0.001, respectively). In contrast, the likelihood of re-sitting for those in the lowest quartile for UMAT-3 at the first test was reduced (OR 0.61, 95% CI 0.57, 0.64, P < 0.001). Increased likelihood of re-sitting the UMAT was predicted by being male (OR 1.10, 95% CI 1.06, 1.14, P < 0.001), being of younger age, being from a non-English language speaking background (Asian language background OR 1.18, 95% CI 1.13, 1.23, P < 0.001), (European language background OR 1.12, 95% CI 1.0, 1.26, P = 0.05) and all Other languages (OR 1.49, 95% CI 1.33, 1.67, P < 0.001), being from NZ (OR 3.59, 95% CI 3.43, 3.76) and being non-ATSI (OR 1.57, 95% CI 1.15, 2.13, P = 0.004). Students from Catholic secondary schools (OR 0.91, 0.86, 0.95, P < 0.001) were less likely to re-sit the UMAT while those from the TAFE school category were more likely (OR 1.67, 1.31, 2.12, P < 0.001). The relative amount of variance explained by the full model was small (6.6%). In a separate model for Australian students only, being rurally based rather than urban was also a significant predictor with rurally based candidates less likely to re-sit (OR, 0.78, 95% CI, 0.71, 0.85, P < 0.001). Finally, logistic regression models were run in those who sat 3 or more times (Table 6) or 4 or more times (Table 7). The pattern of predictor variables for multiple re-sits were largely unchanged in these models, while the relative strength of the odds ratios was increased for each predictor variable together with the relative amount of variance explained by each model.

**Table 5:** Logistic regression for predictors of sitting the UMAT two or more times (N = 17,089) vs once only (N = 110,295) (Nagelkerke R Square 0.066)

***Predictor variable *** **( *reference group in brackets *)**	**N**	**B**	**S.E.**	**Sig.**	**Exp (B)**	**95% CI for exp (B)**	
						**Lower**	**Upper**
***UMAT*-*1 Quartiles *** **( *4 *)**	31971						
UMAT-1 Quartile 1	31853	0.739	0.031	**<0.001**	2.09	1.97	2.23
UMAT-1 Quartile 2	32108	0.712	0.028	**<0.001**	2.04	1.93	2.15
UMAT-1 Quartile 3	31452	0.599	0.026	**<0.001**	1.82	1.73	1.92
***UMAT*-*2 Quartiles *** **( *4 *)**	32340						
UMAT-2 Quartile 1	31685	0.215	0.028	**<0.001**	1.24	1.17	1.31
UMAT-2 Quartile 2	31705	0.250	0.026	**<0.001**	1.28	1.22	1.35
UMAT-2 Quartile 3	31654	0.231	0.026	**<0.001**	1.26	1.12	1.32
***UMAT*-*3 Quartiles *** **( *4 *)**	32939						
UMAT-3 Quartile 1	31346	-0.503	0.027	**<0.001**	0.61	0.57	0.64
UMAT-3 Quartile 2	32166	-0.230	0.025	**<0.001**	0.79	0.76	0.83
UMAT-3 Quartile 3	30933	0-.021	0.024	.374	0.98	0.94	1.03
***Age*** (≤ *16 yr of age*)	1386						
*17 yr*	45316	-0.249	0.076	**0.001**	0.78	0.67	0.91
*18 yr*	55630	-0.400	0.076	**<0.001**	0.67	0.58	0.78
*19 yr*	10787	-0.814	0.081	**<0.001**	0.44	0.38	0.52
*20* - *30 yr*	12203	-0.484	0.080	**<0.001**	0.62	0.53	0.72
>*30 yr*	2062	-0.300	0.101	**<0.001**	0.74	0.61	0.90
***Gender*** (*Females*)	73536						
*Males*	53848	0.096	0.018	**<0.001**	1.10	1.06	1.14
***Language spoken at home*** (*English*)	88134						
*Asian Languages*	34461	0.164	0.020	**<0.001**	1.18	1.13	1.23
*European Languages*	2581	0.116	0.060	**0.05**	1.12	1.00	1.26
*Other Languages*	2208	0.400	0.058	**<0.001**	1.49	1.33	1.67
***School type*** (*Government*)	59827						
*Catholic*	22407	-0.097	0.025	**<0.001**	0.91	0.86	0.95
*Independent*	41812	0.004	0.020	0.829	1.00	0.97	1.05
*Other*	2842	-0.046	0.056	0.411	0.96	0.86	1.07
*TAFE*	496	0.510	0.123	**<0.001**	1.67	1.31	2.12
***Country*** (*Australia*)	109880						
New Zealand	16267	1.279	0.024	**<0.001**	3.59	3.43	3.76
Other Country	1237	0.128	0.094	0.174	1.14	0.95	1.37
***Aboriginal & Torres Strait Islander*** (*ATSI*)	126808						
*Non*-*ATSI*	576	0.450	0.157	**<0.001**	1.57	1.15	2.13

(Significant P-values are boldfaced).

**Table 6:** Logistic regression for predictors of sitting the UMAT three or more times (N = 3307) vs once only (N = 110,295) (Nagelkerke R Square 0.08)

***Predictor variable *** **( *reference group in brackets *)**	**N**	**B**	**S.E.**	**Sig.**	**Exp (B)**	**95% CI for exp (B)**	
						**Lower**	**Upper**
***UMAT*-*1 Quartiles *** **( *4 *)**	28421						
UMAT-1 Quartile 1	28010	1.519	0.075	**<0.001**	4.57	3.94	5.29
UMAT-1 Quartile 2	28252	1.368	0.070	**<0.001**	3.93	3.43	4.51
UMAT-1 Quartile 3	28919	1.032	0.070	**<0.001**	2.81	2.45	3.22
***UMAT-2 Quartiles (4)***	28588						
UMAT-2 Quartile 1	28006	0.331	0.063	**<0.001**	1.39	1.23	1.57
UMAT-2 Quartile 2	28132	0.350	0.060	**<0.001**	1.42	1.26	1.60
UMAT-2 Quartile 3	28876	0.249	0.061	**<0.001**	1.28	1.14	1.45
***UMAT-3 Quartiles (4)***	30032						
UMAT-3 Quartile 1	28000	-0.526	0.058	**<0.001**	0.59	0.53	0.66
UMAT-3 Quartile 2	28208	-0.223	0.054	**<0.001**	0.80	0.72	0.89
UMAT-3 Quartile 3	27362	-0.059	0.052	0.260	0.94	0.85	1.05
***Age*** *(≤ 16 yr of age)*	1208						
*17 yr*	40433	-0.393	0.159	**0.014**	0.68	0.49	0.92
*18 yr*	49719	-0.608	0.159	**<0.001**	0.54	0.40	0.74
*19 yr*	9527	-0.859	0.167	**<0.001**	0.42	0.31	0.59
*20 - 30 yr*	10865	-0.595	0.166	**<0.001**	0.55	0.40	0.76
*> 30 yr*	1850	-0.485	0.209	**0.021**	0.62	0.41	0.93
***Gender*** *(Females)*	65786						
*Males*	47816	0.286	0.037	**<0.001**	1.33	1.24	1.43
***Language spoken at home*** *(English)*	79313			**<0.001**			
*Asian Languages*	30091	0.253	0.041	**<0.001**	1.29	1.19	1.40
*European Languages*	2302	0.121	0.125	0.334	1.13	0.88	1.44
*Other Languages*	1896	0.403	0.113	**<0.001**	1.450	1.20	1.87
***School type*** *(Government)*	52724						
*Catholic*	20367	-0.030	0.052	0.563	0.97	0.88	1.08
*Independent*	37565	-0.034	0.045	0.450	0.97	0.89	1.06
*Other*	2515	0.016	0.108	0.881	1.02	0.82	1.26
*TAFE*	431	0.537	0.241	**0.026**	1.71	1.07	2.75
***Country*** *(Australia)*	99605						
New Zealand	12874	1.542	0.047	**<0.001**	4.67	4.26	5.12
Other Country	1123	-0.151	0.247	0.541	0.86	0.53	1.40
***Aboriginal & Torres Strait Islander*** *(ATSI)*	113061						
*Non-ATSI*	541	0.325	0.322	0.312	1.38	0.74	2.60

(Significant P-values are boldfaced).

**Table 7:** Logistic regression for predictors of sitting the UMAT four or more times (N = 756) vs once only (N = 110,295) (Nagelkerke R Square 0.097)

***Predictor variable (reference group in brackets)***	**N**	**B**	**S.E.**	**Sig.**	**Exp (B)**	**95% CI for exp (B)**	
						**Lower**	**Upper**
***UMAT-1 Quartiles (4)***	27584						
UMAT-1 Quartile 1	27217	2.327	0.187	**<0.001**	10.24	7.11	14.77
UMAT-1 Quartile 2	27603	2.037	0.179	**<0.001**	7.67	5.39	10.89
UMAT-1 Quartile 3	28647	1.330	0.184	**<0.001**	3.78	2.64	5.43
***UMAT-2 Quartiles (4)***	27754						
UMAT-2 Quartile 1	27255	0.320	0.132	**0.016**	1.38	1.06	1.79
UMAT-2 Quartile 2	27555	0.146	0.133	0.273	1.16	0.89	1.50
UMAT-2 Quartile 3	28487	0.164	0.135	0.222	1.18	0.91	1.53
***UMAT-3 Quartiles (4)***	29438						
UMAT-3 Quartile 1	27321	-0.438	0.119	**<0.001**	0.645	0.51	0.82
UMAT-3 Quartile 2	27517	-0.255	0.116	**0.027**	0.775	0.62	0.97
UMAT-3 Quartile 3	26775	-0.022	0.112	0.841	0.98	0.79	1.22
***Age*** *(≤ 16 yr of age)*	1175						
*17 yr*	39603	-0.530	0.313	0.091	0.59	0.32	1.09
*18 yr*	48714	-0.835	0.313	**0.008**	0.43	0.24	0.80
*19 yr*	9191	-1.347	0.331	**<0.001**	0.26	0.14	0.50
*20 - 30 yr*	10563	-0.831	0.326	**0.011**	0.436	0.23	0.83
*> 30 yr*	1805	-0.935	0.428	**0.029**	0.393	0.17	0.91
***Gender*** *(Females)*	64437						
*Males*	46614	0.480	0.076	**<0.001**	1.617	1.39	1.88
***Language spoken at home*** *(English)*	77800						
*Asian Languages*	29175	0.376	0.082	**<0.001**	1.456	1.24	1.71
*European Languages*	2246	0.022	0.274	0.936	1.022	0.60	1.75
*Other Languages*	1830	0.490	0.212	**0.021**	1.633	1.08	2.48
***School type*** *(Government)*	51344						
*Catholic*	19967	0.031	0.106	0.769	1.032	0.84	1.27
*Independent*	36891	-0.019	0.094	0.840	0.981	0.82	1.18
*Other*	418	-0.456	0.259	0.078	0.634	0.38	1.05
*TAFE*	2431	0.868	0.425	**0.041**	2.383	1.04	5.48
***Country*** *(Australia)*	97839						
New Zealand	12101	1.863	0.092	**<0.001**	6.441	5.38	7.71
Other Country	1111	0.380	0.454	0.403	1.463	0.60	3.56
***Aboriginal & Torres Strait Islander*** *(ATSI)*	110517						
*Non-ATSI*	534	0.082	0.583	0.888	1.086	0.35	3.40

(Significant P-values are boldfaced).

## Discussion

This study has identified substantial practice effects for the UMAT which serve to enhance performance, especially between the first and second occasion of testing. The UMAT is now delivered to approximately 15,000 students each year in Australia and New Zealand [2] and hence a more comprehensive appreciation of practice effects will better inform its application in the selection of students for medical and dental schools. Practice effects in cognitive ability tests are now well described, with a meta-analysis [1] estimating that the magnitude of the improvement in test scores from the first test to the first re-sit would be approximately a quarter of the standard deviation from the first test, and a further fifth of a standard deviation to the second re-sit. With respect to high stakes selection such as for medical or dental school selection, re-sitting of admission tests is common. Approximately 40% of students who sat a combination cognitive ability test and science knowledge test for admission to medical schools in Belgium re-sat the test in a 4-year period [9]. Analysis of re-test effects in that study showed a one third of a standard deviation increment in score on the knowledge test and a one half of standard deviation increase for the cognitive ability test. For the UMAT we now report quantitatively comparable results, with an estimated increment at the first re-sit of approximately two fifths of a standard deviation from the first test and a further one fifth of a standard deviation at the second re-sit. For a candidate at the 50^th^ percentile in the first test this would have translated to an increase to the 60^th^ percentile at the second test and to the 63^rd^ percentile at the third test. Our results complement and extend previous reports which have been conducted on a substantially smaller scale [2,4]. Given that 13.5% of students over the 13 years of observation were re-sitting the test these practice effects may be of sufficient magnitude and prevalence to warrant re-consideration in current approaches to medical student selection in Australia and New Zealand.

Practice effects that improve performance in cognitive tests may arise from a number of different potential sources. The authors of the 2012 ACER report on the UMAT [2] favoured issues associated with repetition, such as less confusion because of test preparation and increased familiarity with the test, which may reduce anxiety and improve performance (so called construct irrelevant factors). This understanding of practice effects would lead to the conclusion that the first test score was not a true reflection of the candidate’s ability because of confounding by these factors, and that the construct validity of the test was therefore unchanged on re-testing. However, this cannot be assumed as the entire explanation without further scrutiny. Indeed when item level data were carefully analysed in the cognitive test used for selection of medical students in Belgium [10] it was concluded that re-testing actually led to a change in the measurement properties of the test, such that the predictive validity of the re-test score in relation to academic performance in the first 3 years at medical school was compromised. Evidence for the hypothesis that re-testing and practice effects can alter the psychometric properties and subsequent predictive validity in cognitive tests has been reported by others [11,12] but not by all observers [13]. If practice effects influence the psychometric properties of a test of cognitive ability then the underlying principle of stability of construct relevant variance of the test is violated and calls into question its subsequent predictive validity and utility in selection [13]. Item level analysis for questions delivered in consecutive tests is therefore now required to better delineate the extent to which the practice effects in the UMAT described here reflect construct irrelevant factors versus construct relevant factors.

A separate analysis of the high achieving candidates in the 2012 ACER report [2] identified an actual decrease in UMAT performance rather than an increase, raising the possibility that a substantial proportion of any practice effect may simply represent regression to the mean. This result is similar to the outcome we have seen when comparing candidates in the highest quartile of performance in the UMAT and each of its subsections to those in the lowest quartile. However, when 2 previous studies were re-analysed to better understand the potential influence of regression to the mean on practice effects in cognitive ability tests [1], it was estimated that less than 10% of the practice effect size could be attributed to regression to the mean.

The practice effects identified on re-sitting the UMAT may at least in part represent improvements secondary to either coaching and/or practice between attempts. They may also have been confounded by coaching and/or practice before the first attempt itself which would serve to mitigate any improvement seen at a subsequent attempt. Such coaching in Australia is reported to be as high as 56% of all candidates [3] while for the Medical College Admission Test (MCAT), widely used for medical student selection in North America, a review identified 4 studies in the area where the prevalence of coaching was 22%, 25%, 38% and 72% of candidates respectively [14]. We have not been able to measure such potential influences in this study. However, Griffin et al. [15], in a study of 287 applicants to the School of Medicine at the University of Western Sydney, reported that in the 51.4% of the cohort who had prior UMAT coaching, there was no coaching effect on UMAT-1 and UMAT-2. However, there was evidence of a small coaching effect on UMAT-3 (approximately 3.8% increase in score). This was not significant after adjustment for prior academic performance, age and gender. In a subsequent study [3] they assessed UMAT performance in 402 students from 31 Australian secondary schools who completed the UMAT in their final secondary school year. Again only the UMAT-3 score exhibited an improvement in the 56.2% of the cohort who had been coached, with an increment in mean score of approximately 7.7%. After controlling for prior academic performance, gender and school type, the effect of coaching was again no longer significant. Of interest, however, there was an interaction between prior academic performance and coaching, in that those with higher academic performance performed better with coaching on UMAT-3, while those with weaker academic performance performed worse. Similar results have been reported from New Zealand [4] where a commercial preparation course was shown to have no effect on UMAT-1, UMAT-2 or total UMAT score but UMAT-3 scores increased by 7.1 % in those with no preparation course compared to 16.5% in those who participated in a preparation course. The overall conclusion from these studies is that coaching and practice between sitting and re-sitting the UMAT have had little if no effect on UMAT-1 and UMAT-2 while the effect on UMAT-3 has been relatively small and insufficient to influence the overall UMAT score utilised for medical student selection.

While not always explicitly acknowledged, and not a part of classical test theory, tests of aptitude or proficiency are composed of items differing in difficulty, with the easier items generally at the beginning of a test. The expectation is that the more able students will answer correctly, not only the easier items which the less able students will be able to answer correctly, but in addition, more difficult items which the less able will not be able to answer correctly. Answering the more difficult items, in addition to the easier items, implies a greater understanding on the variable of assessment. In the terminology of Chomsky [16], who distinguished between *competence* and *performance* where the latter is an imperfect indicator or manifestation of the former, not only do the students with a higher score have a better performance, but the higher performance implies greater competence. The practice effects in test administration, especially in longitudinal research design, and perhaps in high stakes testing, have been viewed in the literature as detrimental to the validity of the testing [17]. In particular, using Chomsky’s terminology again, it is implied that the student’s score on a second occasion is inflated in performance relative to the student’s competence. In other more colloquial terms, there has been learning/teaching to the test, rather than improved understanding of the variable to be assessed by the test. Taking the perspective that greater competence implies answering correctly, not only the easy items, but also the more difficult items, this detrimental perspective implies that the effect of practice is to be able to answer the more difficult items, as well as the easier items, without having improved in competence. For the UMAT we identified that the major practice effect was between the first and second test, with a smaller but still significant improvement by the third test. No further improvement was seen with further re-testing. Others who have investigated the nature of practice effects of re-testing in other settings have also observed the largest increment with successive testing is between the first and second occasion [18]. The key observation in that study, however, was that on the second occasion of testing, the increased total score was a result of students answering correctly relatively easy items and not the difficult items. Thus they increased their performance, as evidence by their total score, but not their competence. An increase in performance, rather than competence, has implications for understanding the effects of practice on repeated testing, and further, may have implications for the kind of practice that is provided to ensure that students who sit only once are not disadvantaged relative to those who sit a second time. On the other hand, if not only performance, but apparent competence is improved, then it would indicate that not only has practice eliminated errors in easy items, but that it has permitted answering correctly the more difficult items. This concept needs evaluation through item level analysis of the UMAT, appreciating that the average difficulty of test items has not always been linked to the magnitude of practice effects in other settings [19].

The other confounding factor in understanding practice effects in tests like the UMAT is the background influence of guessing. Waller [20] made the case that guessing on a multiple choice item results from the item being too difficult for the student, and not from some property of the item. Taking this perspective, Andrich et al. [21] studied the effects of guessing by considering that guessing makes difficult items appear relatively easier than if there is no guessing. To study this effect, they were able to eliminate the effect of guessing by a scaling of the items which estimated their relative difficulties. The most surprising result from that study was that it was not so much that the less proficient students, who might have guessed some items correctly, who were advantaged in the original analysis, but that the more proficient students were disadvantaged. This is understood by recognising that the more proficient students answer difficult items correctly at a greater rate than the less proficient students, even when allowing that the less proficient might have guessed. As a result the more proficient students are not rewarded as much as they should be for answering the difficult items correctly. Adjusting the scale for guessing, and then estimating person proficiencies based on all responses of each student, provides a stronger linearisation of the total scores than an original analysis. This more robust approach should also be incorporated in individual item level analysis in the UMAT if further studies of observed practice effects are to more fully elucidate their nature and implications.

A final confounding factor in relation to practice effects may be that applicants who actually choose to re-sit the UMAT are different from those who give up after a single test, or from those who are successfully selected into medical or dental school at their first attempt, and that these differences may have an impact on the magnitude of subsequent practice effects. There have been very few attempts to look at correlates of withdrawal or persistence in relation to multiple re-sits in aptitude tests and none in relation to the UMAT. The single strongest predictor in this study was UMAT percentile score on initial testing. Being male, being younger, being from NZ and being from non-English language backgrounds (although only of borderline significance for those of European language backgrounds) all predicted a greater likelihood of a re-sit even after adjustment for the influence of initial UMAT percentile score. Of interest, it was having a low score in UMAT-1 – Logical reasoning and Problem Solving – that was the single strongest predictor of a re-sit while in contrast, those with a weaker performance in UMAT-3 – Non-verbal Reasoning – were less likely to re-sit. The profile of initial under-performance in UMAT-1 and over-performance in UMAT-3 in those who choose to re-sit recalls the profile we have previously reported in UMAT performance for those from Asian language backgrounds [22]. Given that those from an Asian language background were also nearly 20% more likely to re-sit the test this may have, at least in part, contributed to this finding in a cohort where approximately 27% of the sample reported speaking an Asian language at home. The second strongest predictor was being a student from New Zealand, with a 3.6 fold increase in the likelihood of a re-sit. In New Zealand a larger number of students sit the UMAT twice because entrance into medicine occurs after a one year open entry biomedical course and so they sit both in the last year of secondary school and again in the first year at university [4]. However, our analyses of those who sat four or more times still showed that being from New Zealand continued to be an even stronger predictor, with a 6.4 fold increase in the odds for New Zealanders of 4 or more re-sits. Moreover, the correlates of a re-sit (being male, being from a non-English background, being younger, and being from NZ) were also correlates of a weaker practice effect at the first re-sit, with the exception of being from NZ, which predicted a relatively greater practice effect than being from Australia. Whether this persistence by those from NZ is linked to higher motivation or un-measured socio-cultural differences remains speculative. A contributing factor, however, may be that the 2 medical schools in NZ are both undergraduate entry alone whereas in Australia there are now 12 graduate entry medical programs and so failure to be selected into an undergraduate course may preferentially lead to subsequent sitting of the Graduate Australian Medical Schools Admissions Test (GAMSAT) for graduate school entry rather than repeated re-sits of the UMAT as seen in NZ.

### Study limitations

There are potential limitations to fully understanding the effect of practice from our data. These limitations are associated with having only total percentile scores in the analyses, rather than item level data. First, it is not possible to tell whether the improvement was uniform across the items or whether it was in the easy or difficult items. Second, with multiple choice items, there is a potential for students to guess the difficult items correctly with an impact on the scale of assessment. Guessing also interacts with possible practice effects in the responses that are analysed. Third, the analyses were based on average percentile locations and therefore changes in the percentile locations between years. Because these are therefore relative improvements, it is difficult to assess finer details of the basis for improvement from repeated sitting of the examination. The use of a unique identifier based on name, date of birth and gender to identify subjects who had sat once or more from 2000 to 2012 may have failed to identify some subjects who changed their name over the period. However, these numbers are likely to have been small in this overwhelmingly young cohort of predominantly high school students. Finally, the UMAT has been delivered since the early 1990’s and so given that this data-set was confined to all who sat from 2000 onwards it is possible that for some subjects data analysed as results from the first test during this period may have in fact come from second or subsequent re-sits of the test. This would have if anything weakened the magnitude of the practice effects seen.

## Conclusions

Practice effects of re-sitting the UMAT have been identified in a large cohort of subjects who were candidates for the UMAT between 2000 and 2012. They are of sufficient magnitude to potentially disadvantage those who are sitting the test for the first time. Given the widespread utilisation of the UMAT for selection into medical, dental and allied health courses in Australia and New Zealand such practice effects need further evaluation at the level of individual item level data to ascertain the extent to which they might represent inflated performance or a true increase in competence on re-testing. If the latter, issues of equity in relation to potential increased chances of selection in those who re-sit will be less of a concern, being outweighed by increased confidence that the test is still identifying those most able to enter the health-related professions. If the former, and where UMAT score is utilised as either a threshold factor for subsequent selection for interview or as a core factor in terms of relative weighting with other conventional selection factors (i.e. prior academic performance, score at interview and to a lesser extent a personal statement), any practice effects which serve to enhance overall performance may currently be introducing unwanted bias in selection which should be studied further.

## Competing interests

IP is the representative for the University of Western Australia on the UMAT Consortium Board of Management. AM is a member of the UMAT Test Management Committee and Chair of both the UMAT Technical Subcommittee and the UMAT Research Subcommittee. This study was not commissioned or supported financially by UMAT or ACER.

## Authors’ contributions

IP contributed to the conception and design of the study, acquisition, analysis and interpretation of the data and the initial drafting and final revision of the manuscript. AM contributed to the conception and design of the study, interpretation of the data; and final revision of the manuscript for important intellectual content. DA contributed to the interpretation of the data and the initial drafting and final revision of the manuscript for important intellectual content. IS contributed to the interpretation of the data and to the final revision of the manuscript for important intellectual content. All authors read and approved the final manuscript.

## Pre-publication history

The pre-publication history for this paper can be accessed here:

http://www.biomedcentral.com/1472-6920/14/48/prepub

## Supplementary Material

Additional file 1 **Socio-demographic profile by year the UMAT was first sat 2000-2012.** (The distributions are reported as valid percent exclusive of missing values). Click here for file

## References

[B1] Hausknecht JP Halpert JA Di Paolo NT Meghan O Moriarty G Retesting in selection: a meta-analysis of coaching and practice effects for tests of cognitive ability J Appl Psychol 2007 92 373 385 17371085 10.1037/0021-9010.92.2.373

[B2] Hunt M Pywell S Le L Lay D UMAT2012. Report on the 2012 undergraduate medicine and health sciences admission test A report prepared for the Australian Council of Education Research 2012

[B3] Griffin B Carless S Wilson I The effect of commercial coaching on selection test performance Med Teach 2013 35 295 300 10.3109/0142159X.2012.746451 23228084

[B4] Wilkinson TM Wilkinson TJ Preparation courses for a medical admissions test: effectiveness contrasts with opinion Med Educ 2013 47 417 424 10.1111/medu.12124 23488761

[B5] Mercer A Chiavaroli N UMAT: A validity study. A review of the underlying constructs and an analysis of the content of the undergraduate medicine and health sciences admission test 2006 A report prepared for the UMAT Consortium

[B6] Edwards D Friedman T Coates H Establishing the criterion validity of UMAT: A multi-cohort study of Australian medical students 2011 A report prepared for the UMAT Consortium

[B7] ABS series 1267.0 - Australian standard classification of languages (ASCL) 2011 [http://www.abs.gov.au/ausstats/abs@.nsf/mf/1267.0]

[B8] Measuring remoteness: accessibility/remoteness index of Australia (ARIA) revised edition. Occasional papers: new series Number 14 2001 [http://www.health.gov.au/internet/main/publishing.nsf/Content/health-historicpubs-hfsocc-ocpanew14a.htm]

[B9] Lievens F Buyse T Sackett PR Retest effects in operational selection settings: development and test of a framework Pers Psychol 2005 58 981 1007 10.1111/j.1744-6570.2005.00713.x

[B10] Lievens F Reeve CL Heggestad ED An examination of psychometric bias due to retesting on cognitive ability tests in selection settings J Appl Psychol 2007 92 1672 1682 18020804 10.1037/0021-9010.92.6.1672

[B11] Hausknecht JP Trevor CO Farr JL Retaking ability tests in a selection setting: implications for practice effects, training performance, and turnover J Appl Psychol 2002 87 243 254 12002953 10.1037/0021-9010.87.2.243

[B12] Te Nijenhuis J Voskuijl OF Schijve NB Practice and coaching on IQ tests: quite a lot of *g* Int J Sel Assess 2001 9 302 308 10.1111/1468-2389.00182

[B13] Reeve CL Lam H The psychometric paradox of practice effects due to retesting: measurement invariance and stable ability estimates in the face of observed score changes Intelligence 2005 33 535 549 10.1016/j.intell.2005.05.003

[B14] McGaghie WC Downing SM Kubilius R What is the impact of commercial test preparation courses on medical examination performance? Teach Learn Med 2004 16 202 211 10.1207/s15328015tlm1602_14 15294461

[B15] Griffin B Harding DW Wilson IG Yeomans ND Does practice make perfect? The effect of coaching and retesting on selection tests used for admission to an Australian medical school MJA 2008 189 270 273 18759725 10.5694/j.1326-5377.2008.tb02024.x

[B16] Chomsky N Aspects of the theory of syntax 1976 Cambridge, Massachusetts: MIT Press

[B17] Casey MB Brabeck MM Exceptions to the male advantage on a spatial task: family handedness and college major as factors identifying women who excel Neuropsychologia 1989 27 689 696 10.1016/0028-3932(89)90113-9 2739890

[B18] Andrich D Styles I Psychometric evidence of intellectual growth spurts in early adolescence J Early Adolesc 1994 14 328 344 10.1177/0272431694014003002

[B19] Powers DE Relations of test item characteristics to test preparation/test practice effects: a quantitative summary Psychol Bull 1986 100 67 77

[B20] Waller MI Modeling guessing behavior: a comparison of two IRT models Appl Psychol Meas 1989 13 233 243 10.1177/014662168901300302

[B21] Andrich D Marais I Humphry S Using a theorem by Andersen and the dichotomous Rasch model to assess the presence of random guessing in multiple choice items J Educ Behav Stat 2012 37 417 442 10.3102/1076998611411914

[B22] Puddey IB Mercer A Carr SE Louden W Potential influence of selection criteria on the demographic composition of students in an Australian medical school BMC Med Educ 2011 11 97 10.1186/1472-6920-11-97 22111521 PMC3233506

