# Supplementary material for: Practice effects in medical school entrance testing with the undergraduate medicine and health sciences admission test (UMAT)
Source: BMC Med Educ. 2014 Mar 12;14:48. doi: 10.1186/1472-6920-14-48 (PMC4007585; doi:10.1186/1472-6920-14-48)
Supplement: Additional file 1 — Socio-demographic profile by year the UMAT was first sat 2000-2012. (The distributions are reported as valid percent exclusive of missing values). [file 1472-6920-14-48-S1.docx]

**Additional File 1** Socio-demographic profile by year the UMAT was first sat 2000-2012. (The distributions are reported as valid percent exclusive of missing values).

| ***Year First Sat Umat***  ***(N)*** | 2000  (6540) | 2001  (5766) | 2002  (7088) | 2003  (8168) | 2004  (9471) | 2005  (10492 | 2006  (11925) | 2007  (12760) | 2008  (12589) | 2009  (13116) | 2010  (13433) | 2011  (12677) | 2012  (11808) |
| --- | --- | --- | --- | --- | --- | --- | --- | --- | --- | --- | --- | --- | --- |
| ***Age*** |  |  |  |  |  |  |  |  |  |  |  |  |  |
| *≤ 16 yr* | 1.3% | 1.3% | 1.4% | 1.3% | 1.4% | 1.1% | 1.2% | 1.0% | 1.0% | 1.0% | 0.9% | 1.0% | 1.1% |
| *17 yr* | 35.2% | 40.3% | 40.2% | 37.4% | 35.9% | 36.0% | 34.7% | 34.5% | 34.4% | 35.1% | 35.2% | 34.2% | 34.6% |
| *18 yr* | 37.8% | 39.2% | 40.9% | 41.8% | 40.3% | 41.8% | 43.4% | 43.3% | 45.3% | 44.5% | 45.1% | 46.4% | 46.1% |
| *19 yr* | 6.8% | 5.3% | 4.7% | 7.3% | 9.6% | 9.1% | 8.9% | 10.0% | 8.7% | 8.7% | 8.8% | 8.5% | 8.1% |
| *20 - 30 yr* | 15.9% | 11.5% | 11.0% | 10.4% | 10.9% | 10.5% | 10.0% | 9.6% | 9.0% | 9.1% | 8.5% | 8.5% | 8.6% |
| *> 30 yr* | 3.0% | 2.4% | 1.9% | 1.8% | 2.0% | 1.5% | 1.7% | 1.5% | 1.5% | 1.6% | 1.5% | 1.4% | 1.5% |
| ***Gender*** |  |  |  |  |  |  |  |  |  |  |  |  |  |
| *Females* | 59.1% | 60.7% | 59.9% | 58.7% | 59.3% | 58.0% | 57.3% | 57.5% | 58.1% | 57.6% | 57.3% | 55.5% | 56.4% |
| *Males* | 40.9% | 39.3% | 40.1% | 41.3% | 40.7% | 42.0% | 42.7% | 42.5% | 41.9% | 42.4% | 42.7% | 44.5% | 43.6% |
| ***Language spoken at home*** |  |  |  |  |  |  |  |  |  |  |  |  |  |
| *English* |  | 73.9% | 73.0% | 71.6% | 69.3% | 69.8% | 68.1% | 71.0% | 70.6% | 68.8% | 67.7% | 66.8% | 64.7% |
| *Asian Languages* |  | 17.8% | 19.4% | 23.9% | 26.6% | 26.7% | 26.7% | 26.4% | 27.0% | 28.4% | 29.6% | 31.0% | 32.3% |
| *European Languages* |  | 2.7% | 2.2% | 2.4% | 2.2% | 2.6% | 1.0% | 2.1% | 2.0% | 2.0% | 2.0% | 1.7% | 1.7% |
| *Other Languages* |  | 5.6% | 5.4% | 2.1% | 1.9% | 0.9% | 5.3% | 0.5% | 0.5% | 0.8% | 0.7% | 0.5% | 0.8% |
| ***School Type*** |  |  |  |  |  |  |  |  |  |  |  |  |  |
| *Government* |  | 41.4% | 41.8% | 44.5% | 46.1% | 46.3% | 48.1% | 48.6% | 48.2% | 48.5% | 48.3% | 48.3% | 46.6% |
| *Catholic* |  | 20.7% | 19.5% | 18.7% | 16.9% | 17.0% | 17.5% | 17.1% | 17.8% | 17.0% | 17.8% | 16.6% | 17.4% |
| *Independent* |  | 36.2% | 36.7% | 34.4% | 33.9% | 33.7% | 31.7% | 31.3% | 31.4% | 31.9% | 31.4% | 32.6% | 33.4% |
| *Other* |  | 1.3% | 1.6% | 2.1% | 2.5% | 2.6% | 2.4% | 2.5% | 2.2% | 2.3% | 2.2% | 2.2% | 2.2% |
| *TAFE* |  | 0.4% | 0.4% | 0.4% | 0.5% | 0.4% | 0.4% | 0.5% | 0.4% | 0.4% | 0.3% | 0.3% | 0.3% |
| ***Country*** |  |  |  |  |  |  |  |  |  |  |  |  |  |
| *Australia* | 98.5% | 98.1% | 97.8% | 91.0% | 84.7% | 85.6% | 85.2% | 84.3% | 84.8% | 84.4% | 83.4% | 83.3% | 86.4% |
| New Zealand | 1.1% | 1.4% | 1.7% | 8.3% | 14.2% | 13.0% | 13.5% | 14.5% | 14.4% | 14.7% | 15.8% | 15.7% | 12.6% |
| Other Country | 0.3% | 0.5% | 0.5% | 0.7% | 4.0% | 1.4% | 1.3% | 1.2% | 0.8% | 0.9% | 0.8% | 0.9% | 1.0% |
| ***Aboriginal & Torres Strait Islander*** |  |  | 0.4% | 0.5% | 1.3% | 1.0% | 0.3% | 0.4% | 0.2% | 0.3% | 0.3% | 0.4% | 0.4% |
